# sEMG-Based Hand Gesture Recognition Using Binarized Neural Network

**DOI:** 10.3390/s23031436

**Published:** 2023-01-28

**Authors:** Soongyu Kang, Haechan Kim, Chaewoon Park, Yunseong Sim, Seongjoo Lee, Yunho Jung

**Affiliations:** 1 School of Electronics and Information Engineering, Korea Aerospace University, Goyang-si 10540, Republic of Korea; 2 Department of Information and Communication Engineering, Sejong University, Seoul 05006, Republic of Korea; 3 Department of Convergence Engineering of Intelligent Drone, Sejong University, Seoul 05006, Republic of Korea; 4 Department of Smart Air Mobility, Korea Aerospace University, Goyang-si 10540, Republic of Korea

**Keywords:** surface electromyography, hand gesture recognition, spectrogram, binarized neural network, field-programmable gate array

## Abstract

Recently, human–machine interfaces (HMI) that make life convenient have been studied in many fields. In particular, a hand gesture recognition (HGR) system, which can be implemented as a wearable system, has the advantage that users can easily and intuitively control the device. Among the various sensors used in the HGR system, the surface electromyography (sEMG) sensor is independent of the acquisition environment, easy to wear, and requires a small amount of data. Focusing on these advantages, previous sEMG-based HGR systems used several sensors or complex deep-learning algorithms to achieve high classification accuracy. However, systems that use multiple sensors are bulky, and embedded platforms with complex deep-learning algorithms are difficult to implement. To overcome these limitations, we propose an HGR system using a binarized neural network (BNN), a lightweight convolutional neural network (CNN), with one dry-type sEMG sensor, which is implemented on a field-programmable gate array (FPGA). The proposed HGR system classifies nine dynamic gestures that can be useful in real life rather than static gestures that can be classified relatively easily. Raw sEMG data collected from a dynamic gesture are converted into a spectrogram with information in the time-frequency domain and transferred to the classifier. As a result, the proposed HGR system achieved 95.4% classification accuracy, with a computation time of 14.1 ms and a power consumption of 91.81 mW.

## 1. Introduction

Human–machine interfaces (HMI), which use advanced methods without traditional interface equipment such as a keyboard, mouse, and touch screen, are widely developed in fields such as robotics, augmented reality (AR)/virtual reality (VR), and prosthetic control [1,2,3]. Among them, hand gesture recognition (HGR) systems are popular because they use hand gestures that intuitively represent a user’s intention.

Sensors used in HGR systems can be divided into vision-based and non-vision-based sensors. The camera, a representative vision-based sensor, relies on the acquisition environment, uses a large amount of data, and does not easily adapt to frequent location changes by users. In particular, because of external factors such as background and lighting changes, very complex detection algorithms are required. In the case of the inertial measurement unit (IMU), which is a representative non-vision-based sensor, a glove-type sensor is widely used to detect finger movements [4]. The IMU sensor has the advantage of being able to acquire hand movements most intuitively; however, the glove-type sensor can limit activity when worn.

An electromyography (EMG) sensor, a non-vision-based sensor, measures the EMG signal—a bio-signal. The EMG signal is measured by amplifying the micro-electric signal generated by signal transmission between the nerves and muscle fibers when the muscle contracts and includes intuitive muscle activity information [5]. Methods for acquiring EMG signals include invasive and non-invasive methods. Invasive EMG (iEMG) is a method of measurement by inserting a needle into the skin. Surface EMG (sEMG) acquires data through electrodes attached to the skin. The electrodes used to obtain the sEMG are divided into wet and dry types. The wet electrode uses a conductive gel to reduce noise and stably acquire data. However, a conductive gel must be used each time a sensor is worn, and the life of the electrode is short. Dry-type sensors can conveniently acquire data by touching the electrode to the skin.

Recently, dry-type sEMG sensors have been widely applied to sEMG-based HGR systems because of their advantages of being able to obtain information on hand and finger movement, by attaching them to the forearm or wrist, and only a small amount of data being required. Some previous studies have shown excellent factors in overall system development, but studies on suitability for real-life applications are insufficient. From the user’s point of view, we assume the following constraints to ensure comfort when the HGR system is applied in real life: (1) It is easy to wear and take off and should not affect daily life when wearing it. Therefore, it is preferable to use a small number of dry-type sensors. (2) The computational time between gesture and recognition should not exceed 100 ms so that the user can recognize it as a real-time system [6]. (3) Wearable systems are usually battery-based, so power consumption should be low. (4) The HGR system should classify gestures that can correctly communicate the user’s intentions to the machine (e.g., gestures that are less likely to be made unconsciously, predefined dynamic gestures). Additionally, the number of gestures should be appropriate. (5) Finally, the classification accuracy should be greater than 95%.

The studies [7,8,9,10,11,12,13,14,15,16,17,18,19,20] implemented an sEMG-based HGR system on CPUs and GPUs, taking advantage of their computational capabilities and versatility. The authors of [8,13,17,18] achieved more than 95% classification accuracy, but [13] presented the results of classifying relatively few simple static gestures. The studies [8,17,18] used CNN-based complex classifiers such as ResNet-50, and [17] had an average computation time of 465 ms, which is unsuitable for real-time systems. In addition, CPU- and GPU-based systems generally consume a lot of power. To overcome these CPU and GPU limitations, Refs. [4,21,22,23,24,25,26,27,28,29,30] implemented systems on a microcontroller unit (MCU) or field-programmable gate array (FPGA). However, due to embedded systems’ characteristics, it is difficult to implement complex algorithms due to limited resources. The studies [4,21,22,23,24,25,26,27,28,29,30] used machine learning algorithms that use simple computational processes. These studies used multiple sensors or classified fewer and simpler gesture classes to overcome the limitations of machine learning, which is fast and simple but has a lower performance than deep learning algorithms.

In this paper, we propose an HGR system that acquires sEMG data using only one wrist-worn dry-type sensor to satisfy the aforementioned constraints of the HGR system. The sEMG signal obtained from the gesture operation contains information on the gesture in the time and frequency domains. Therefore, the proposed HGR system converts the data acquired from the sensor into a spectrogram via short-time Fourier transform (STFT), transfers it to the classifier, and then classifies the gesture according to the result. The proposed HGR system is implemented on an FPGA for low power consumption and utilizes a deep learning algorithm, a binarized neural network (BNN), to classify nine complex dynamic gestures with data obtained through one sensor. Because a BNN is a lightweight convolutional neural network (CNN) in which the input of each layer and the weight of the kernel are binarized into 0 and 1, it can be implemented on an FPGA with limited resources. The main contribution of this paper can be summarized as follows: (1) Using only one dry-type sEMG sensor, we classified nine complex dynamic gestures with a high accuracy of 95.4%. (2) By implementing the proposed HGR system on an FPGA, a low power consumption of 91.81 mW, and computational time of 14.1 ms were achieved, satisfying the real-time system conditions.

The remainder of this paper is organized as follows: Section 2 reviews the background knowledge of STFT, CNN, and BNN, which are algorithms applied to the proposed HGR system. Section 3 describes the overview of the proposed HGR system, the gestures to classify, data acquisition, pre-processing, and network configuration and evaluation. Section 4 describes the structural design of the proposed HGR system implemented in hardware. Section 5 presents the hardware implementation results of the proposed HGR system and the comparison results between the previous sEMG-based HGR system and the proposed HGR system. Finally, Section 6 presents the conclusions and future research.

## 2. Background

### 2.1. Short-Time Fourier Transform

Fast Fourier transform (FFT) is a signal processing technique that converts data in the time domain into data in the frequency domain. If the FFT result is calculated for the entire given time-domain data, information about the time domain disappears. The STFT is used to overcome the limitations of the FFT. In STFT operation, time domain data are segmented at regular intervals, multiplied by a window function, and then FFT is performed. The result of the STFT operation of each segment contains frequency information, and because the frequency information changes along the index of the segment, time information can also be obtained. Two-dimensional data consisting of information in the time-frequency domain are expressed as an image called a spectrogram. The STFT equation is expressed by the following equation, Equation (1):

(1) $$Y\left(m,f\right)=\int_{-\infty }^{\infty }x\left(t\right)\omega \left(t-m\right)e^{-j2\pi ft}dt$$

where x(t) is the input signal, ω is the window function, and *m* is the window delay time.

### 2.2. Convolutional Neural Network

**Figure 1:**
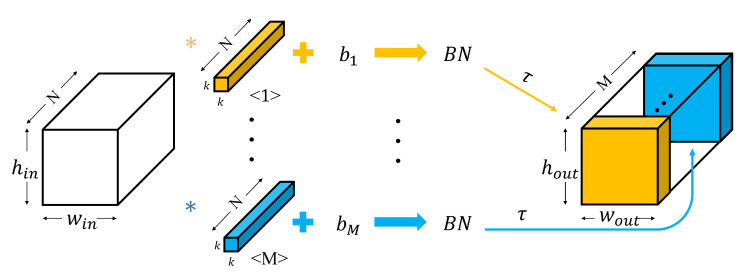
Process of the convolution layer.

Deep learning algorithms, which do not require a separate feature extraction process and have the advantage of high classification performance, are used in various fields. Among them, CNN, which extracts features through convolution with the kernel while maintaining the dimension of the input data, has attracted attention. A CNN consists of convolution layers and fully connected layers. In the convolution and fully connected layers, weight convolution or multiplication, bias addition, batch normalization (BN), and activation function are applied to the input data. In the convolution layers, these processes are performed using a 2-dimensional matrix, and the input/output data are called feature maps. In the fully connected layers, these processes are performed using a 1-dimensional vector, and the input/output data are called nodes. The process of the convolution layer is shown in Equation (2):

(2) $$X_{j}^{n}=\tau \left(BN((\sum_{i\in N,j\in M}X_{i}^{n-1}*K_{ji}^{n})+b_{j}^{n})\right)$$

where $X_{j}^{n}$, $K_{ji}^{n}$, and $b_{j}^{n}$ represent the *j*th feature map output by the *n*th layer convolution layer, convolution kernel corresponding to input/output feature map, and bias, respectively. *N* and *M* are the number of input and output feature map channels, respectively. τ, *BN*, and * are the activation function, batch normalization, and convolution operation, respectively. Figure 1 shows the process of the convolution layer. *k* is the size of the convolution kernel.

### 2.3. Binarized Neural Network

Generally, CNN’s feature maps, nodes, and weights are composed of several bits of floating-point data. CNN also involves complex computations such as multiplication and division in convolution and BN. Therefore, CNN requires a significant amount of memory to store many learned parameters and feature maps, and complex arithmetic circuits are essential for the calculation process. Thus, it is not easy to implement in hardware with limited resources. A BNN is a lightweight CNN that overcomes these limitations. The BNN’s feature maps, nodes, and weights are composed of +1 and −1 instead of multi-bit floating-point data, and can be expressed as 1-bit data. Therefore, the product of binarized data can be replaced by the XNOR operation. Because the results of BN are binarized to +1 or −1 through the activation function, BN and activation function can be replaced by comparing convolution results with pre-trained thresholds.

## 3. Proposed HGR System

**Figure 2:**
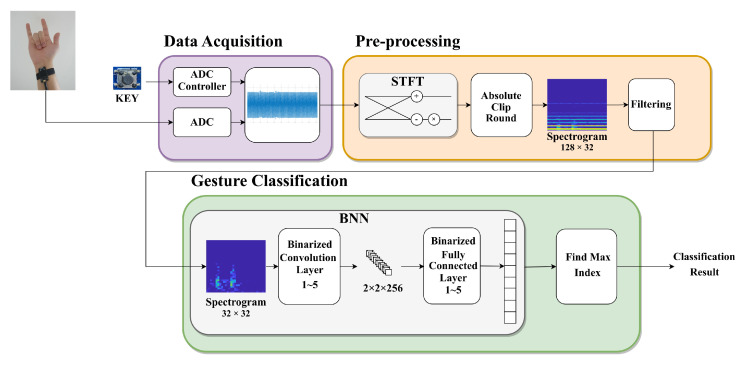
Overview of the proposed HGR system.

The proposed HGR system acquires the sEMG signal generated by the user’s gesture with one sensor, pre-processes it, and then transfers the pre-processed data into a pre-trained neural network to classify the user’s gesture. An overview of the proposed HGR system to be implemented in hardware is shown in Figure 2. The user’s sEMG signal was transferred from the sensor to an analog-to-digital converter (ADC) built into the FPGA and converted into digital data. The converted data were pre-processed through STFT and converted into a spectrogram. The absolute values were taken, and classification was performed by transferring them into the BNN.

### 3.1. Gestures Definition

**Figure 3:**
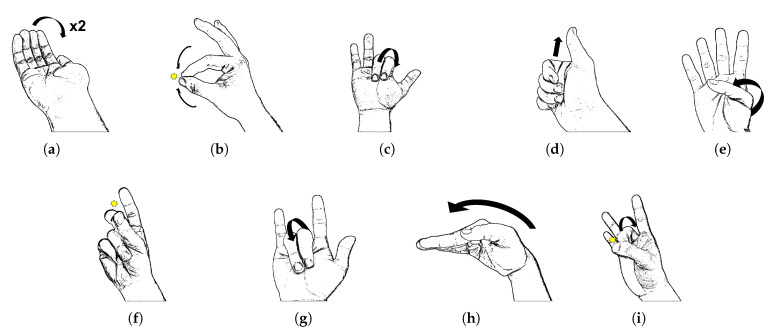
Hand gesture examples: (**a**) Gesture 1; (**b**) Gesture 2; (**c**) Gesture 3; (**d**) Gesture 4; (**e**) Gesture 5; (**f**) Gesture 6; (**g**) Gesture 7; (**h**) Gesture 8; (**i**) Gesture 9.

Hand gestures can be classified into static and dynamic gestures. Static gestures do not involve hand movements during data acquisition, whereas dynamic gestures involve hand movements. We chose to classify dynamic gestures for practical use. Gestures consist of a rest–motion–rest process and include dynamic movements of fingers and wrist. We selected nine gestures, as shown in Figure 3.

Gesture 1 is the motion of folding and unfolding of four fingers, excluding the thumb, twice. Gesture 2 is the motion of pinching the index finger and thumb together. Gesture 3 is the motion of folding and unfolding the index and middle fingers. Gesture 4 is the motion of bending the thumb into the other fingers, with the fingers curled, and then raising the thumb. Gesture 5 is the motion of folding and unfolding the thumb into the palm with the other fingers straight. Gesture 6 is the motion of snapping the thumb against the middle finger. Gesture 7 is the motion of bending the middle and ring fingers into the palm and slightly turning the wrist outward. Gesture 8 is a 90-degree inward bending of the wrist. Gesture 9 is an upward flicking motion of the middle finger against the thumb.

### 3.2. Data Acquisition

**Figure 4:**
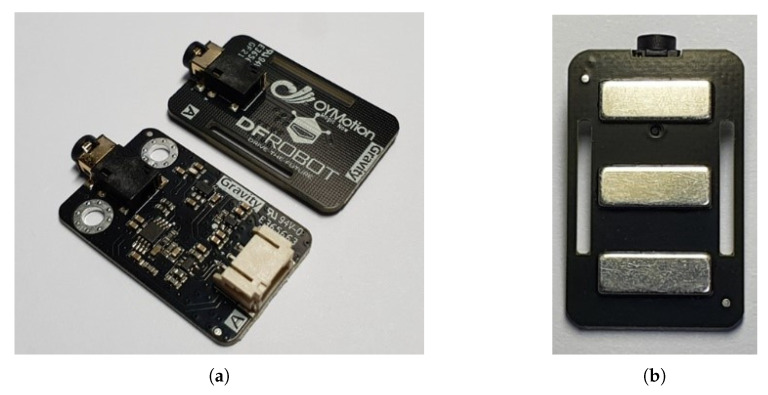
sEMG sensor used in the proposed HGR system: (**a**) configuration of sensor; (**b**) front view of electrode module.

The sEMG sensor used in the proposed HGR system was Gravity [31], as shown in Figure 4. Figure 4a shows two modules of the sensor. The upper part is a module with electrodes, and the lower part is a module that includes signal-processing circuits, such as signal amplification. When this module is brought into contact with the desired body part, the electrical signal generated by that part can be detected. The proposed HGR system specified the inner wrist for electrode placement. Because most of the sEMG signals concentrate in the frequency band between 10 and 500 Hz [32], the FPGA sampled the user’s sEMG signal at 1000 Hz for 2.112 s and converted it into 10-bit digital data.

### 3.3. Pre-Processing

Because the sEMG data acquired during static gestures do not vary significantly in characteristics over time, it is adequate to extract time-domain features with pre-processing in terms of speed and implementation complexity. However, to classify complex dynamic gestures, the pre-processing results must include more information about the gestures. From this perspective, time-frequency domain analysis can achieve a better system performance than time or frequency domain analysis alone. For example, Ref. [19] showed that using a spectrogram to classify many dynamic gestures achieves a better classification accuracy than using the root mean square (RMS). The spectrogram is a fundamental component of the time-frequency distribution in the analysis of signals, particularly for noise and artifact reduction [33]. Therefore, we generated a spectrogram using STFT in the pre-processing.

**Figure 5:**
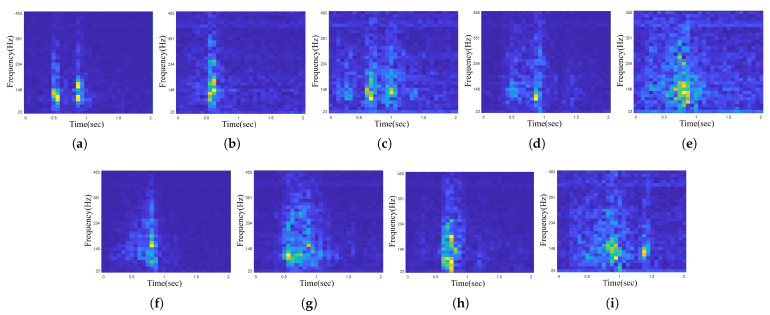
Spectrograms of nine gestures: (**a**) Gesture 1; (**b**) Gesture 2; (**c**) Gesture 3; (**d**) Gesture 4; (**e**) Gesture 5; (**f**) Gesture 6; (**g**) Gesture 7; (**h**) Gesture 8; (**i**) Gesture 9.

The proposed HGR system obtained 2112 raw data points from ADC. The spectrogram was generated using a 128-point FFT, which corresponds to a window length of 128 ms, and Hamming window. With an overlap ratio of 50%, a 128 × 32-sized spectrogram was obtained. The frequency axis length is 128, and the time axis length is 32. Because the input data are real numbers, the FFT results are symmetrical around the DC. The obtained sEMG signal has a hum noise of 60 Hz, and this noise appears in addition to frequency band multiples of 60 Hz. To remove this noise, we removed two to six adjacent frequency components at DC, 60 Hz, 120 Hz, 180 Hz, 240 Hz, 300 Hz, 360 Hz, 420 Hz, and 480 Hz, respectively, from the FFT results. Only 32 values were selected through a filtering method that removes noise-containing frequency components from 64 positive frequency data, including DC. Consequently, a spectrogram with a size of 32 × 32 was generated. Figure 5 shows the spectrogram after filtering for each gesture.

The spectrogram is an image that represents the power distribution in the time-frequency domain of a signal. It consists of the absolute values of STFT results, which are complex numbers. Because the proposed HGR system was implemented in hardware, it was necessary to determine a method for calculating the absolute value. The square and root operations of Equation (3) require complex circuits to be implemented in hardware.

(3) $$\left|x\right|=\sqrt{ℜ{\left(x\right)}^{2}+ℑ{\left(x\right)}^{2}}$$

In the proposed HGR system, the system was simplified using Equation (4), which is a much simpler absolute value calculation method, although it results in an error compared to the result of Equation (3).

(4) $$\left|x\right|=\left|ℜ(x)\right|+\left|ℑ(x)\right|$$

The size of the calculation result of Equation (4) is one to two times larger than the theoretical value. However, there is little difference in performance from implementation using Equation (3), and the hardware complexity is much lower. Table 1 presents the performance comparison results of the two methods.

### 3.4. Performance Evaluation with Network

Experiments were conducted in a software environment to evaluate the network performance. Prior to the experiments, sEMG raw data were acquired to obtain a spectrogram dataset to be used in the experiment. The sEMG signal may exhibit slightly different characteristics even with the same gesture, depending on the user, the sweat on the skin where the sensor is attached, the position of the sensor, and the degree of fatigue of the user. Therefore, for accurate experimental results, we collected sEMG data from four people, acquired three times a day for 13 days. Thus, we obtained unbiased data for several factors that could change the sEMG signal characteristics.

Before constructing the BNN, the CNN, which is the basis of the BNN, was selected through various experiments. Approximately 14,400 experimental data points were used, with approximately 1600 per gesture. A total of 11,600 data points, approximately 80% of the total data, were used as the training data. The remaining 2800 data points were used as the test data. Experiments were conducted by changing the number of layers, number of filters, and number of nodes. The adaptive moment estimation (Adam) optimizer and cross-entropy function were used during training. The epoch was set to 200, the batch size was 128, and the learning rate was set to 0.01, 0.005, 0.0005, 0.00005, and 0.000005 for each epoch of 0, 40, 80, 120, and 160, respectively.

Table 2 lists the performance of each network. All tested networks include a max pooling layer in the convolution layer, except for the first convolution layer, and all convolution layers include zero padding. In general, there is a trade-off between the accuracy and complexity of CNN. Therefore, Network 1 with reasonable complexity and accuracy was chosen for the BNN experiment. The memory usage of the weights in the selected network is 6,317.184 KB when storing each value as full-precision floating-point data.

Experiments were conducted to design a BNN with excellent performance. Among the evaluated networks, five networks with high accuracy were tested as BNN. The results are summarized in Table 3. Parameters include weights and thresholds.

**Figure 6:**
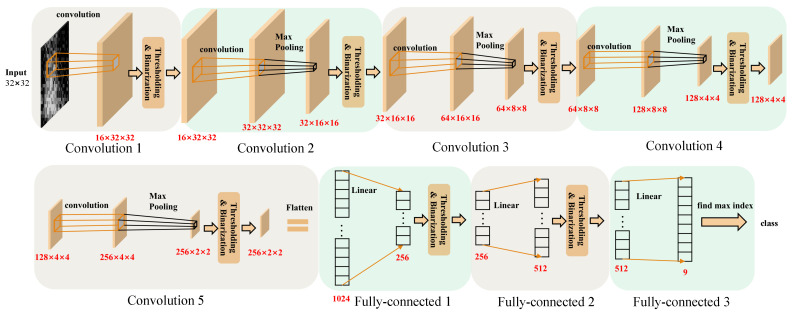
Architecture of BNN.

Finally, a BNN with a structure using five convolution layers and three fully connected layers was selected as shown in Figure 6. All layers of the network contain thresholding and binarization, which are batch normalization and activation functions. The weights of the selected network use 98.706 KB of memory, which is a 98.4% reduction compared with the memory usage of the CNN network.

## 4. Hardware Architecture Design

**Figure 7:**
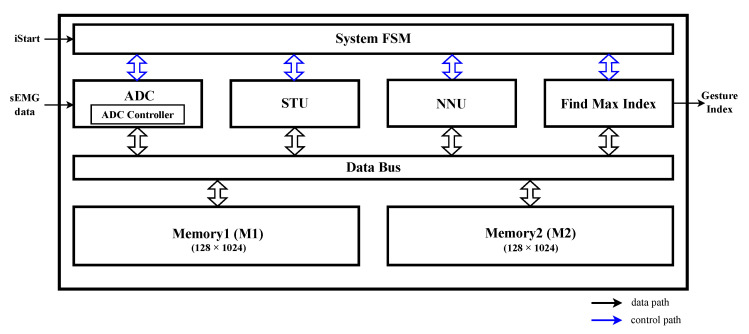
Block diagram of the proposed HGR system.

The proposed HGR system consists of an ADC unit, STFT unit (STU), BNN unit (NNU), two memory units to be used as buffers, and a data bus that manages communication between the operation units and buffers, as shown in Figure 7. The proposed HGR system utilizes an ADC unit built into an FPGA. Memory1 (M1) and Memory2 (M2) are used by the STU and NNU in a ping-pong scheme. The ADC unit stores sEMG signals in M1 according to a sampling period of 1000 Hz. Every time a certain number of raw data are accumulated, pre-processing is performed, and the spectrogram is completed at the end of 32 STFTs. The NNU uses the spectrogram as an input and outputs nine final node values. For example, if the fifth value among the nine output values is the largest, the input data are classified into the fifth class. In other words, the class is determined using a simple algorithm that finds the index of the node with the highest value among the results of NNU.

### 4.1. STFT Unit

**Figure 8:**
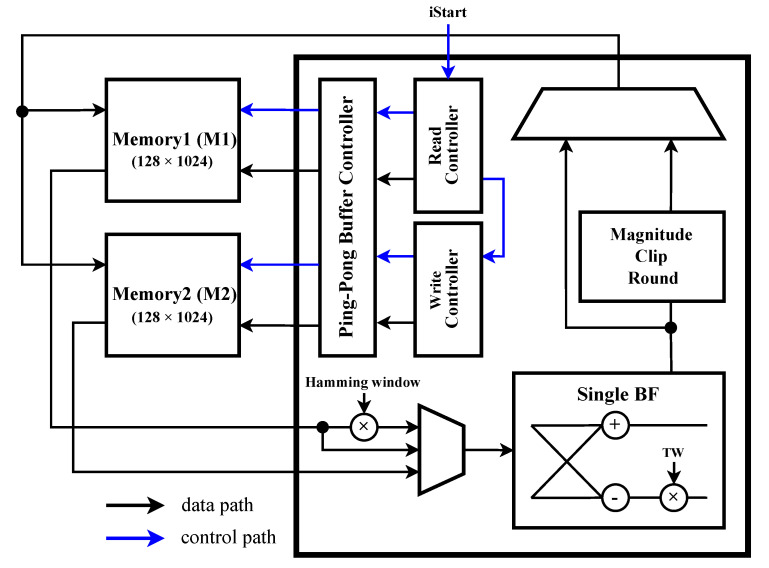
Block diagram of the STFT unit.

A block diagram of the STU is shown in Figure 8. In Figure 8, the data bus between the STU and the memory is omitted. For each STFT, memories and STU exchange appropriate data through the Ping-Pong Buffer Controller. The ADC raw data are multiplied by the Hamming window, then transferred to a single butterfly (BF), and the stage 1 operation is performed and stored in M2. Then, based on the result of stage 1 stored in M2, the stage 2 operation is performed, and the result is stored in M1. In this way, M1 and M2 are used alternately, an operation up to stage 7 is performed, and the final result of the STFT is stored in M1. After STFT is performed 32 times, NNU starts classification using the completed spectrogram.

### 4.2. BNN Unit

**Figure 9:**
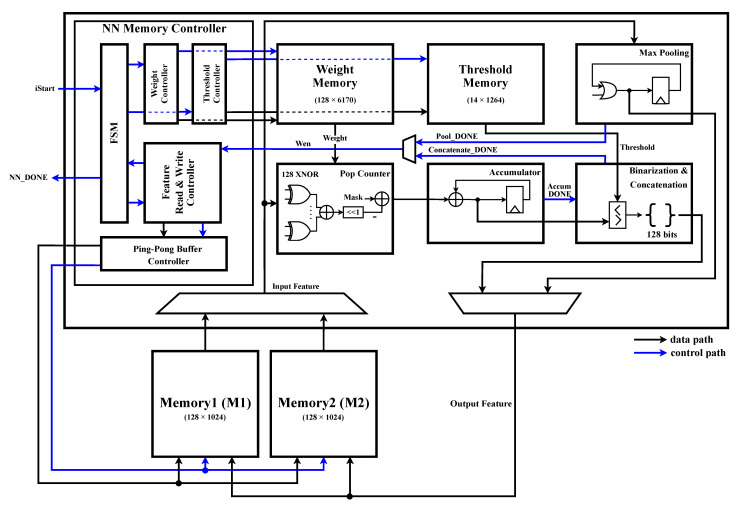
Block diagram of the BNN unit.

A block diagram of the NNU is shown in Figure 9. In Figure 9, the data bus between the NNU and the memory is omitted. When the STU finishes generating the spectrogram, the iStart signal, which is a start signal to the NNU, is transferred to the finite state machine (FSM). The FSM generates a state that controls the NNU operation. The FSM outputs the CONV state of the convolution layer, POOL state of the max pooling layer, and FCL state of the fully connected layer. In the CONV state, the memory controller fetches the appropriate input feature maps, weights, and thresholds. The XNOR operation results of the feature map and weight are accumulated through the Pop Counter. The result is accumulated through the Accumulator up to the size of the kernel. Then, in the Binarization and Concatenation, the accumulated value is compared with the threshold, and 1-bit data binarized by the number of output channels are concatenated and stored in memory. The input and output feature maps are stored in M1 and M2 in the ping-pong scheme through the Ping-Pong Buffer Controller. In the POOL state, the size of the feature map is reduced through max-pooling. Because all the pixel data of the feature map are binarized 1-bit data, the process of finding the maximum value is simplified to an OR operation. The FCL state performs the same process as the CONV state. However, the difference is that in the CONV state, the feature map is a 2-dimensional matrix, and in the FCL state, the nodes are a 1-dimensional vector. That is, in the CONV and FCL states, the operations of generating the address and enabling the signal of the memory controller are different, whereas the other operations are the same.

## 5. Hardware Implementation Results

The proposed HGR system was designed using the Verilog hardware description language (HDL) and implemented on an Intel-Altera MAX 10 10M50DAF484C7G FPGA [34]. The proposed HGR system operated at a clock frequency of 50 MHz. It consumed 1.83 mW of dynamic power and 89.98 mW of static power. Table 4 shows the number of logic elements, registers, and digital signal processors (DSPs) used in the STU and NNU, and the other circuits, such as ADC, ADC controller, and filtering.

Table 5 shows the memory usage of the proposed HGR system. M1 and M2 require a width of 128 for an XNOR parallel operation and a depth of 1024 to store a spectrogram of 32 × 32. In the STU, the Hamming window requires a width of 8 and a depth of 128 for a 128-point FFT. The twiddle factors require a width of 10 and a depth of 64. In addition, in the NNU, a depth of 6170 is required to store 789,648 1-bit data with a width of 128, and a width of the thresholds is experimentally selected as 14.

Table 6 shows the number of clock cycles used during one test at the STU and NNU and the computation time for a 50 MHz clock frequency. Because the sEMG signals have a time interval of 1 ms and one STFT operation uses a time of approximately 21 µs for a 50 MHz clock, more than one STFT operation is possible between two sEMG signals. Therefore, 32 STFTs to generate a spectrogram can be performed while all 2,112 sEMG signals are acquired. As a result, the inference process by NNU for one gesture takes 14.1 ms after the sEMG signal acquisition is finished.

**Figure 10:**
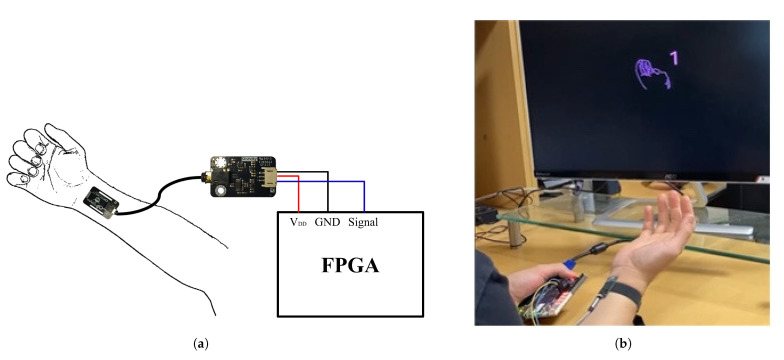
Experimental environment: (**a**) example of sensor being worn; (**b**) experimental environment with display.

Figure 10a shows an example of wearing a sensor, and Figure 10b shows the actual experimental environment on the FPGA platform, with the classification result displayed on the monitor. In the experiment, an accuracy of approximately 98% was recorded in 100 trials on the FPGA platform, and real-time operation was confirmed.

As mentioned above, the previous sEMG-based HGR systems have been implemented on various platforms, including CPU, GPU, MCU, and FPGA. However, CPU- and GPU-based systems typically consume several watts to tens of watts, making them difficult to integrate into wearable devices. Therefore, a comparison between the proposed HGR system and the sEMG-based HGR systems implemented with the MCU and FPGA is presented in Table 7. In general, as the number of gestures to be classified increases, the classification process becomes more complex and the computation time increases. Therefore, this study presents the computation time divided by the number of classes. We intensively analyzed the papers presented in Table 7 on the conditions of the HGR system that can be applied to real life, as mentioned in the introduction.

As seen from Table 7, except for the proposed HGR system, all studies used two to 64 sensors. The studies [21,22,23,25,26,27] used wet-type sensors. Among them, that of [26], where the wearing location is the wrist, consumes very little power; however, the classification gestures (Rest, Open, Grasp, Pronation, and Supination) are very simple, and the computation time exceeds 100 ms. Study [24] shows a fast computation time using the fewest sensors except for the proposed HGR system, but as in [26], classification gestures (Hand closing, Hand opening, Wrist flexion, Wrist extension, and Double wrist flexion) are very simple and the classification accuracy is lower than the proposed HGR system. The studies [29,30] had a higher classification accuracy and faster computation time than the proposed HGR system. However, Ref. [29] used sensors with a single drop of conductive gel for 64 dry electrodes. The study [30] used a two-slot adhesive skin interface for each sensor, and classified five very simple static gestures (Hand close, Thumb close, Thumb-index, Middle-ring, and Middle-ring-little). In addition, both systems implemented in the FPGA consume more power than the proposed HGR system. Both [4,28] had low classification accuracy, and used many sensors. The proposed HGR system classified nine dynamic gestures with a high classification accuracy of over 95% using a single dry-type sEMG sensor. The computation time of the proposed HGR system was slower than that of some previous studies because of the deep learning algorithm, which is more complex than machine learning. However, it satisfies the 100 ms criterion, which is a condition of the real-time system presented in [6], and achieved a classification accuracy of more than 95% and low power consumption of 91.81 mW.

## 6. Conclusions

In this study, we propose an HGR system that classifies nine dynamic gestures based on a dry-type sEMG sensor and a BNN suitable for hardware design. To achieve high classification accuracy with only one sEMG sensor, a spectrogram including time-frequency domain information was generated by STFT in pre-processing and classified with a deep learning algorithm. To overcome the limitations of deep learning algorithms with high complexity and a long computation time, BNN, which is a lightweight CNN, was used. Owing to its lightening, it was successfully implemented in low-cost FPGA. The FPGA design enabled the design of a fast computation time and low power consumption, and ultimately, it was able to satisfy the conditions of the HGR system to be applied in real life. Some previous studies are superior to the proposed HGR system in terms of the classification accuracy, computation time, and power consumption. However, the proposed HGR system is very useful for classifying nine dynamic gestures using only one sensor, with a classification accuracy of 95.4%.

The limitation of this study is that there is still room for development in terms of computation time and power consumption. In future work, we plan to implement an sEMG-based HGR system in very large-scale integrated (VLSI) with faster computation time, and less power consumption.

## Figures and Tables

**Table 1:** Accuracy according to the absolute value calculation method and logic elements usage of absolute value calculation unit.

Implementation Method	Accuracy (%)	Logic Elements
Equation (3)	95.6	662
Equation (4)	95.4	61

**Table 2:** Accuracy of CNNs by number of filters and nodes.

Network	#filters					#nodes				Accuracy (%)
	C^1^1	C^1^2	C^1^3	C^1^4	C^1^5	F^2^1	F^2^2	F^2^3	F^2^4	
1	16	32	64	128	256	256	512	9	–	97.4
2	16	32	64	128	–	256	512	9	–	96.3
3	16	32	64	128	256	512	512	9	–	96.3
4	16	32	64	128	256	256	512	512	9	97.8
5	16	32	64	128	256	512	1024	9	–	96.6
6	16	32	64	128	256	256	256	1024	9	96.7
7	16	32	64	128	256	256	256	512	9	97.1

^1^ Convolution layer; ^2^ fully connected layer.

**Table 3:** Accuracy and parameters of BNNs based on CNNs with excellent performance.

Network	#filters					#nodes				Accuracy (%)	Parameters
	C^1^1	C^1^2	C^1^3	C^1^4	C^1^5	F^2^1	F^2^2	F^2^3	F^2^4		
1	16	32	64	128	256	256	512	9	-	95.4	790,912
4	16	32	64	128	256	256	512	512	9	95.5	1,053,568
5	16	32	64	128	256	512	1024	9	-	94.9	1,451,648
6	16	32	64	128	256	256	256	1024	9	94.8	992,896
7	16	32	64	128	256	256	256	512	9	95.0	856,704

^1^ Convolution layer; ^2^ fully connected layer.

**Table 4:** Implementation results of the proposed HGR system.

Unit	Logic Elements	Registers	DSPs
STU	960	234	36
NNU	1938	374	10
Others	679	263	-
Total	3577	871	46

**Table 5:** Memory usage of the proposed HGR system.

Memory	Width	Depths	Memory Usage (bits)
M1	128	1024	131,072
M2	128	1024	131,072
Hamming window	8	128	1024
Twiddle factors	10	64	640
Weights	128	6170	789,760
Thresholds	14	1264	17,696
Total	-	-	1,071,264

**Table 6:** Computation cycles and time for the proposed HGR system.

Unit	Computation Cycles	Computation Time (µs) (@ 50 MHz Clock Frequency)
STU	1053	21.06
NNU	704,930	14,098.6

**Table 7:** Comparison results between the proposed HGR system and the previous sEMG-based HGR systems implemented on MCU and FPGA.

Ref.	Platform	Sensor			Classification				Implementation Results		
		Type	Wearing Position	#Sensors	#Classes	Gesture Type	Classifier	Accuracy (%)	Computation Time	Computation Time/#Class	Power (mW)
[21]	ARM	wet	forearm	4	4	static	SVM ^1^	83.9	2.2 ms	625 µs	n/a
[22]	RISC-V	wet	forearm	8	11	static	HDC ^2^	85	36 µs	3.27 µs	10.4
[23]	ARM	wet	forearm, wrist	8	7	static	SVM ^1^	89.2	1 ms	0.14 ms	86
[24]	ARM	dry	forearm	2	5	static, dynamic	SVM ^1^	92	10 ms	2 ms	n/a
[25]	ARM	wet	forearm	4	10	static, dynamic	ANN ^3^	94	0.2 ms	20 µs	100.6
[26]	ARM	wet	wrist	4	5	static, dynamic	SVM ^1^	94	250 ms	50 ms	5.1
[27]	ARM	wet	forearm	8	6	n/a	LDA ^4^	94.14	n/a	n/a	122.4
[4]	FPGA	dry	forearm	16	12	static	GBDT ^5^	90.7	n/a	n/a	n/a
[28]	FPGA	dry	forearm	8	9	static	KNN ^6^	93.4	n/a	n/a	n/a
[29]	FPGA	dry with gel	forearm	64	13	static	HDC ^2^	97.12	236.32 µs	2.02 µs	141.2
[30]	FPGA	dry with interface ^7^	forearm	8	5	static	SVM ^1^	98	322 µs	64.3 µs	3,100
Proposed	FPGA	dry	wrist	1	9	dynamic	BNN	95.4	14.1 ms	1.57 ms	91.81

^1^ Support vector machine, ^2^ Hyperdimensional computing, ^3^ Artificial neural network, ^4^ Linear discrimination analysis, ^5^ Gradient boosting decision tree, ^6^ K-nearest neighbors, ^7^ Dry-type sensors with adhesive skin interface.

## Data Availability

Not applicable.
